# *Sarcocornia neei* as an Indicator of Environmental Pollution: A Comparative Study in Coastal Wetlands of Central Chile

**DOI:** 10.3390/plants7030066

**Published:** 2018-08-17

**Authors:** Verónica Meza, Camilo Lillo, Daniela Rivera, Eva Soto, Rodrigo Figueroa

**Affiliations:** 1 Facultad de Ingeniería, Universidad de Playa Ancha, Av. Carvallo 270, Valparaíso, Chile; camilo.lillo@upla.cl (C.L.); daniela.rivera@upla.cl (D.R.); esoto@upla.cl (E.S.); 2 Instituto de Geografía, Pontificia Universidad Católica de Valparaíso, Valparaíso, Chile; rodrigo.figueroa@pucv.cl

**Keywords:** *Sarcocornia* sp, coastal wetlands, heavy metals analysis

## Abstract

Being adapted to saline environments, halophytes are plant species that have received considerable attention due to their ability to cope with environmental stress factors, such as high concentrations of soluble salts and heavy metals. In this work, we focused on determining if the *Sarcocornia neei* (*S. neei*) plant can be considered as an indicator of heavy metal pollution in soil. This was done by analyzing the concentration of cadmium (Cd), lead (Pb), copper (Cu), and arsenic (As) in plants and soil sampled from two wetlands in the central zone of Chile: a wetland contaminated by industrial activities and a wetland protected by the Chilean government. In addition, 14 fertility parameters (pH, electrical conductivity, organic matter, nitrogen (N), phosphorus (P), potassium (K), sodium (Na), Pb, calcium (Ca), magnesium (Mg), Manganese (Mn), zinc (Zn), iron (Fe), and boron (B)) were analyzed for soil samples in both wetlands. This was done to differentiate between available elements and contamination by heavy metals. Plant and soil samples in the contaminated wetland exhibited significantly higher heavy metal concentrations in comparison to samples analyzed from the protected wetland. This indicates that the *S. neei* plant can be further researched as an indicator of heavy metal pollution in saline soils and possibly for phytoremediation purposes.

## 1. Introduction

Coastal wetlands are extremely sensitive ecosystems that represent the interface between terrestrial and marine environments. Wetlands are recognized for being an integral part of larger landscapes and for providing ecosystem services of great importance. Some of these are the retention and removal of nutrients, carbon sequestration, provision and remediation of water quality, etc. [1]. Due to these ecosystem services, wetlands have been categorized as environments of significant ecological complexity and are hence relevant for conservation [2].

In the central zone of Chile, there exists a wetland system with more than 20 wetlands, and these wetlands have different types of hydrology and land use. Some of these wetlands present similar vegetation, where the native plant species *Sarcocornia neei* (*S. neei*), which does not have conservation problems, is found. *S. neei* is presented as a halophytic meadow plant that reaches coverings of 100% and does not exceed 50 centimeters in height (for more information about the taxonomy of *S. neei*, see Reference [3]). *S. neei* is considered to be an important plant and has been studied for different applications, for example, as an alternative forage crop for sheep [4], and also for the effect of salinity on its germination [5]. Other species of that genus, *Sarcocornia,* have been widely studied. For example, *Sarcocornia perennis* (*S. perennis*) has been exposed to different sea water concentrations to research its physiological responses [6]; *Sarcocornia fructicosa* (*S. fructicosa*) has been studied for its productivity and nutritional value [7]; *Sarcocornia ambigua* has been investigated for its process of germination [8]; and *Sarcocornia quinqueflora* has been included in an assessment of environmental pollution in coastal wetlands [9]. Lutts and Lefèvre (2015) [10] performed a literature review that demonstrated that *S. fructicosa* and *S. perennis* exhibit tolerance to high levels of heavy metals, which makes them promising candidates for phytoremediation of heavy metal pollution. Other species of that genus, different from *S. neei,* have been used as indicators of heavy metal contamination [11].

The species of *Sarcocornia* grow grouped in soils with saline characteristics generally near the coast all over the world, except for East Asia. Some of these are restricted to arid continental areas, such as on the shores of salt lakes and marshes, and even in basins between high mountain ranges. In particular, *S. neei* is distributed in saline soils along the Pacific coast of South America from Peru to Chile and in the lowlands of Argentina, with the exception of the Strait of Magallanes [3]. This suggests the possible development of *S. neei* in coastal areas with climatic similarity in which the plant grows naturally.

We studied the *S. neei* plant in two coastal wetlands in central Chile. The first coastal wetland of interest in this study, called Los Maitenes, is less than 4 km away from a strategic industrial seaport located in the Puchuncaví Valley. It is made up of more than 14 companies and mostly includes refineries and thermoelectric power plants. This industrial complex has existed since 1964, and in 1992, the Chilean government enacted a regulation policy that introduced new pollution controls and abatement technologies to improve the air quality in the area. The policy managed to reduce emissions below the permissible limit according to Chilean law [12]. Nevertheless, over the years, new companies have settled in the area, creating a scenario that severely affects human health [13] and causes environmental stress. The latter is evident in the level of contamination by heavy metals in the soil and water matrix, ultimately affecting the ecosystem of the area [14,15,16]. The second coastal wetland of this study is a lagoon called Matanzas, which forms part of the El Yali basin. With its 11,500 ha, it encompasses about 14 different bodies of water. More than 4% of the total area (approximately 520 ha) is protected by Chilean law [17] as a national reserve and, since 1996, as a Ramsar site by the convention with the same name [18], which is an intergovernmental treaty that provides the framework for national action and international cooperation for the conservation and wise use of wetlands and their resources.

The aim of this study is to establish if *S. neei* is an accurate indicator of environmental pollution. To accomplish this, we compared the levels of heavy metals in the soil and plants in two coastal wetlands located in Central Chile. 

## 2. Materials and Methods 

This section outlines the materials and methods that describe the process of collecting the samples through validated protocols, both for soil and for the *S. neei* plant, as well as the statistical methods that were applied.

### 2.1. Study Area

**Figure 1:**
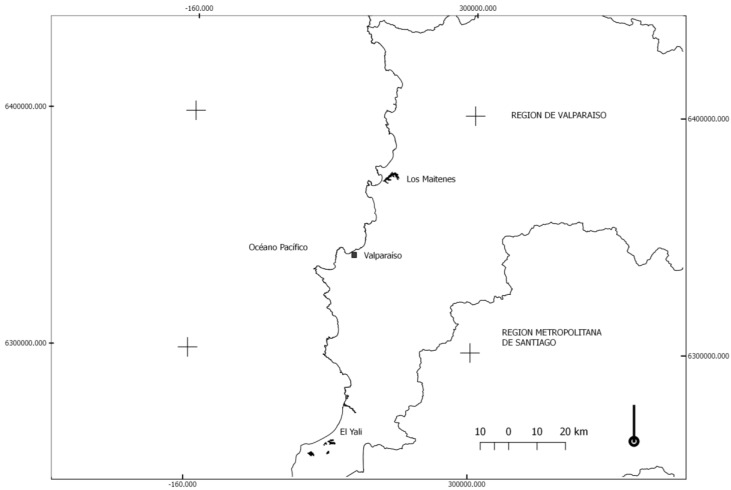
Central zone of Chile and coastal wetlands of interest in black. In the same line of the Pacific Ocean, at a distance of approximately 150 km.

Los Maitenes coastal wetland is located at latitude 320°45′S and longitude 710°28′W, close to a rural location with fewer than 200 habitants. It is less than 4 km away from a strategic industrial area and seaport composed of foundries and companies that generate energy from natural gas and coal. Moreover, Los Maitenes belongs to the wetland systems in south-central Chile, formed by the coastal plain catchment. Matanzas lagoon is located at latitude 330°49′S 330°43′S and longitude 710°46′W 710°22′W. Figure 1 shows the location and distance between the coastal wetlands.

### 2.2. Soil and S. neei Analysis

**Figure 2:**
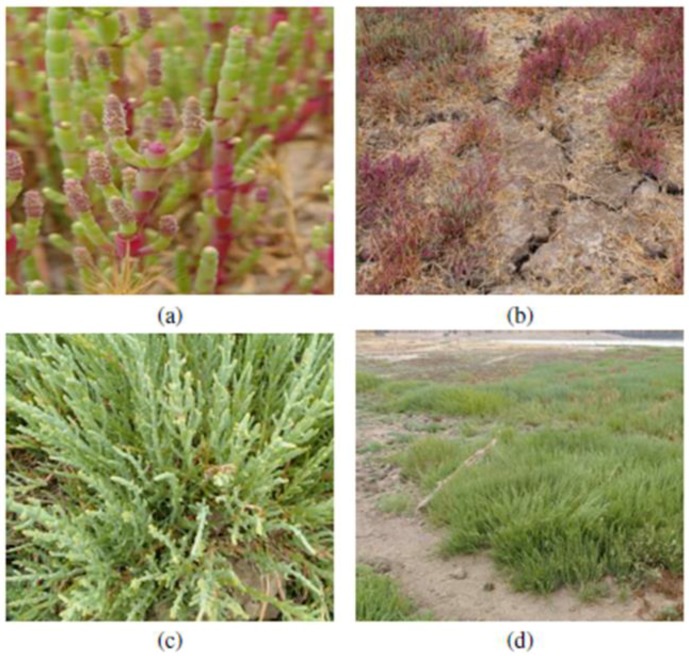
State and morphology of *S. neei* in Los Maitenes (**a**,**b**) and Matanzas (**c**,**d**) coastal wetlands. You can see a difference in color. Note the difference in color of *S. neei* leaves.

For both bodies of water, we used a soil sampling protocol validated by the Ministry of Agriculture of Chile, based on the work of Swift and Sparks [18]. The methodology involved the identification of homogenous zones associated with soil type, the presence of clusters, slope, vegetation coverage, and the dismissal of roads or animal passages of regular use. Soil samples were collected at a depth of 20 cm and mixed in a clean container to acquire a composite sample, from which the amount of 1 kg of soil was separated for laboratory analysis at a certified soil testing laboratory. Stones or clusters were not included in the study. We used several methods to determine the fertility parameters (the fertility is a soil quality indicator; the macronutrients are nitrogen (N), phosphorus (P) and potassium (K); the pH and EC are not nutrients, but depending on their value they allow the bioavailability of the nutrients for the plants in general), which are briefly outlined as follows: The saturation extract method was used to estimate the pH and electrical conductivity (EC); wet combustion and colorimetric determination of reduced chromate was used for organic matter (OM) and extraction with KCl_2_N; NH_4_^+^ and NO_3_^−^ levels were determined by distillation; the Olsen method was employed to determine the available N; and the separation of P and exchangeable K was determined using NaHCO_3_ 0.5 N and an ammonium acetate solution, respectively. For the determination of heavy metals such as cadmium (Cd), lead (Pb), and copper (Cu) in the soil and plants, nitric acid digestion and perchloric acid by spectrophotometry by direct aspiration was used. Note that for Arsenic (As), nitric acid digestion with perchloric acid was used and determined with a hydride generator, in accordance with the procedures recommended for soils in Chile [19] and the methods of analysis of sludge and soils [20]. The plants were selected using a transect method [21]. The extraction of *S. neei* was performed through transects in the southern area of the Matanzas lagoon, which corresponds to a protected area. A total of 20 transects were carried out transversely to the edge of the lagoon, each with an extension of 12–15 m, separated by a length of 2 cm between them, where each point of extraction took place every 50 cm. The criteria for the selection of plants were to consider older specimens, or plants that reached 50 cm in height, with abundant presence of leaves and without alterations due to pests or diseases. The plants were extracted from the root and later moved to the laboratory in clean containers. Prior to heavy metal concentration analysis, the specimens were rinsed with water to eliminate superficial dirt. For each transect, a number of 10 to 15 plants were extracted. The sampling area at Los Maitenes was performed at the east side (parallel to the body of water). The transects at this area were 60 m long with a 1.5 m separation between them. The extraction point was every 2 m with the same plant selection criteria as in the Matanzas lagoon. An average of 20 plants were obtained for each transect at this sampling area. Figure 2 shows plants of *S. neei*, found in both coastal wetlands.

### 2.3. Statistical Analysis

The data were tested for normality using a Shapiro–Wilk (SW) test. The significant difference between the heavy metal concentrations in the plants of the two wetlands was determined using an unpaired t-test and a Mann–Whitney U test, as appropriate. We used a significance level of 5%. The 14 fertility parameters were characterized, grouping them into different components, with a principal component analysis (PCA) [22]. The implementation of the statistical methods was performed in SPSS software, version 23 [23].

## 3. Results

The trace elements formed a group of chemical elements that are found in the environment in minimal quantities. Although plants often use some of them for their nutrition, such as iron (Fe), Cu, and zinc (Zn), among others, most can be harmful to the environment and human health when they occur at higher levels. Particularly dangerous are mercury (Hg), Cd, and As. Table 1 presents the concentration of heavy metal in the soil and plants (n = 5 for each coastal wetland).

From Table 1, note that there are significant differences between both coastal wetlands for the plants (*p* < 0.05 in the Mann–Whitney U test) for Cd, Pb, Cu, and As. In addition, for Cd and Pb concentrations, there are no significant differences between the plants and the soil in Los Maitenes.

Table 2 presents the concentrations of the fertility parameters (n = 15 for each coastal wetland). In the Maitenes wetland, there is a higher concentration of fertility parameters compared to the Matanzas lagoon. Specifically, P, Na, Ca, Zn, Cu, and B in Los Maitenes have concentrations greater than Matanzas, while pH (can be expressed as: hydrogen ion concentration), N, K, manganese (Mn), and Fe in Los Maitenes have concentrations lower than Matanzas. EC, OM, Na, Mn, and Fe do not show significant differences between the two wetlands. Note that variability is also increased in the same elements that are greater in Los Maitenes coastal wetland. In addition, we found that with significance level of 5% all fertility parameters are distributed normally.

Table 3 shows four principal components, for which the joint variability of these four components is 88.09%. Table 4 shows the composition of each component greater than 0.4.

## 4. Discussion

According to the work of Schalscha and Ahumada [24], for more than six decades, there has been concern for the environmental sanitation of the coastal dryland where Los Maitenes coastal wetland is located. In Table 1 and Table 2, high levels of Cu can be observed (in an exploratory way) in the soils of Los Maitenes coastal wetland.

Dust and gases emitted from smelter industries have contaminated soils and plants in varying degrees depending on the distance from the sampling area to the pollution site. Other contributing factors are elevation and the direction of the wind, which create an unfavorable scenario for air quality and consequently for the health of the population, vegetation, and soil [25]. Hence, studies have shown that surface soils near copper or lead smelters have arsenic concentrations ranging from 260 to 380 mg/kg as well as levels of contamination that cause or imply contents of approximately 60–80 mg/kg of As in plants [26]. Thus, from Table 1, there are no problems related to the As level.

In general, for the *S. neei* plant, we detected higher levels of metals in the sample from Los Maitenes coastal wetland, showing absorption of high concentrations of heavy metals, giving this plant possible phytoremediation properties [27] for cleaning the contaminated soil of coastal wetlands. The data can be compared to another study performed in 2004 in central Chile, where two species, *Baccharis linearis* and *S. obtusiloba*, presented levels of 535 mg/kg and 679 mg/kg, respectively [28]. If we compare this with the Spanish Royal Decree 1310/1990, where values are differentiated by pH level and the limit of Cd is 1 mg/kg and is 50 mg/kg for both Pb and Cu, all levels are exceeded in Los Maitenes. Note that Matanzas presents levels of Cd, Cr, and Pb lower than the permitted limit.

It is clear from investigations carried out in the area of Puchuncaví, in the year 2008 [29], related to Cu metal, that there would be accumulating species of metal in its aerial biomass. At least 22 pseudometalofitas species have been found. The species were classified according to their Cu concentration and showed mostly an average (200–600 mg kg^−1^) or low (<200 mg/kg) Cu accumulation. The species with the highest concentration of Cu was *Oenothera affinis* (614 mg/kg). The authors point out that it is necessary to verify that there was no overestimation of the Cu concentrations in the plants, due to the adhesion of polluting particles in the trichomes. The limitations of this study are related to investigating only one metal, with the processing of the plants before determining the concentration and not evaluating the initial level of the soil. In our research we worked in exchange with propagated plants, whose origin is a free zone of metal in the soil. In Chañaral Bay, 800 km north of the study area, the Salicornia SP plant was studied. In addition, its level of metal absorption the results of absorption shown in Table 5. It should be mentioned that Sarcocornia and Salicornia belong to the taxonomic complex of highly specialized plants in continental and coastal saline habitat [30].

The PCA showed that the main elements consist of the micronutrients B, Zn, Cu, and Fe and the macronutrient P, which are available for plants in the soil as orthophosphates, which are soluble inorganic forms since they are monobasic (H_2_PO_4_^−^) and dibasic (HPO_4_^2−^) ions [32]. The five elements were found in neutral to slightly basic pH, which determines their availability for plants and leaching in the soil. B is absorbed in the form of boric acid (B(OH)_3_), or borate (B(OH)^4−^), at neutral and alkaline pH; the availability in the soil solution is higher at pH 5–7 and decreases to pH 7.5–8.5 [33]. According to the second component of the PCA, the relationship between the EC and the Na^+^ is observed, particularly in Zone C of Los Maitenes wetland, where the Na^+^ reaches 70.0 cmol/kg. The salt in high concentrations exerts an osmotic effect which results in decreased water absorption capacity [34]. The soils affected by Na^+^ and other salts are common in arid, semiarid, subhumid, or humid regions where the annual precipitation is insufficient to meet the evapotranspiration needs of the plants. As a result, the salts found in the soil do not dissolve. Instead, an accumulation of salts in the vegetations occurs. For component 3, soil nutrients (N, P, and K) are present, and for micronutrients and trace elements, Mn is present in a greater proportion to the component. Four components have Ca, Mn, and pH. Note that there is a high level of Cu available and is at a greater level in the soils of Los Maitenes than in the two soils examined in the Matanzas. In the case of Fe, the availability for established crops in calcareous soils is low, causing Fe deficiency in plants and soil productivity.

The species *S. neei* grows to a similar height in both wetlands. This could be an indicator that contamination does not affect the growth of this plant species. (For more information about the taxonomy of *S. neei*, see Reference [3]). It would be advisable to study the behavior of the roots of the species in soils with and without pollution, making comparisons of fine root biomass, fine root length and tissue density among other variables [35]. The latter could relate the level of pollution in different areas with the morphological characteristics of the studied species.

## 5. Conclusions

This work presents a comparison of heavy metal concentrations of *S. neei* in two coastal wetlands located in Central Chile. We based our study on samples of the soil and plants and obtained fertility parameter concentrations in the soil and values of heavy metals in the *S. neei* plant.

*S. neei* had remarkable differences in the concentrations of heavy metals, coinciding with the concentrations of these in the soil studied from Los Maitenes. However, the growth of the *S. neei* plant was not affected, and the visual differences were manifested through color variation. *S. neei* plants were not affected by the high level of Na at Los Maitenes coastal wetland. In addition, there were high concentrations of heavy metals, associated with Cu refineries, and the concentrations found exceeded the maximum allowable limit under Spanish regulations. We conducted a PCA to study the relationships that exist between the variables that correlate soil fertility with the elements present in the soil, noting that the first component is represented by B, P, Cu, Zn, and Fe. These components show chemical absorption similar to salts at a neutral to slightly alkaline pH.

Therefore, *S. neei* could be a potential plant for cleaning heavy metal soils in coastal wetlands because it absorbs large concentrations of heavy metals, since it accumulates them and grows even in soils with high levels of Na and Cu.

This research could be used as a pilot study for a further investigation that considers the methodology used to compare fertility parameters in different geographical areas. The results obtained in this investigation are of interest to environmental and/or governmental, national, or foreign agencies and can be used to determine the impact of pollution produced by large industries related to copper refineries, thermoelectric plants, or chemical companies.

## Figures and Tables

**Table 1:** Concentration of heavy metals in the soil and plants (mg kg^−1^ ± SE).

Metal	Soil Los Maitenes *	Soil Matanzas *	*S. neei* Los Maitenes	*S. neei* Matanzas
Cd	2.88 (±0.54)	1.06 (±0.37)	2.7 (±0.77)	<0.01 (±0.08)
Pb	26.70 (±10.24)	17.80 (±8.01)	25.40 (±11.84)	<0.01 (±0.10)
Cu	523.30 (±98.13)	17.90 (±3.45)	332.00 (±62.28)	10.20 (±3.28)
As	61.23 (±14.25)	5.38 (±0.69)	3.07 (±0.58)	0.09 (±0.12)

* Three composite samples were extracted with 6 sub-samples each at a depth of 20 cm.

**Table 2:** Exploratory data analysis for the fertility parameters.

Fertility Parameters *	Unit	Average Concentrations ± SE Los Maitenes Matanzas		*t*-Test *p*-Value	*SW* *p*-Value
pH	25 °C	7.6 (±0.06)	7.71 (±0.11)	<0.01	0.36
CE	ds/m	55.21 (±15.42)	24.95 (±1.94)	0.42	0.11
MO	%	4.48 (±0.55)	4.19 (±0.87)	0.11	0.73
N	mg/kg	9.57 (±0.38)	11.21 (±1.69)	0.01	0.52
P	mg/kg	60.40 (±12.09)	26.90 (±1.00)	0.01	0.35
K	mg/kg	297.67 (±18.03)	564.50 (±60.67)	0.03	0.23
Na	cmol/kg	33.30 (±3.76)	11.02 (±1.34)	0.23	0.09
Ca	cmol/kg	32.43 (±7.71)	23.05 (±2.95)	0.04	0.86
Mg	cmol/kg	11.27 (±1.22)	15.59 (±1.74)	0.04	0.09
Zn	mg/kg	15.63 (±2.56)	1.01 (±0.529)	0.01	0.51
Mn	mg/kg	29.19 (±2.73)	25.80 (±4.36)	0.15	0.53
Fe	mg/kg	210.36 (±153.21)	64.75 (±8.50)	0.35	0.16
Cu	mg/kg	130.40 (± 25.55)	4.70 (±0.80)	0.01	0.49
B	mg/kg	10.94 (±0.71)	1.36 (±0.31)	0.04	0.82

* Three composite samples were extracted with 6 sub-samples each at a depth of 20 cm.

**Table 3:** Eigenvalues and explained variance using PCA.

Components	Total	Percentage of Variance	Cumulative Percentage of Variance
1	5.51	39.37	39.37
2	4.24	30.31	69.68
3	2.57	18.41	88.09
4	1.66	11.90	100.00
5	<0.01	<0.01	100.00

**Table 4:** Structure of principal components.

	1	2	3	4
B	0.97			
P	0.93			
Zn	0.9			
Cu	0.9			
Fe	0.81			−0.77
Na		0.98		
EC		0.96		
N		−0.87	0.53	
MO			0.95	
K			0.85	
Mg			0.73	
Ca				0.87
Mn			0.55	−0.82
pH	−0.62	−0.69		0.7

**Table 5:** Metal concentration in soil and plant in Chañaral Bay, north of Puchuncaví, at 800 km, on the same line of the Pacific.

Metal	Soil Bay of Chañaral (mg/kg)	* *Salicornia* Sp. (mg/kg)
Cu	946	182.6
Fe	249,000	4557
Mn	185	135.6
As	<12.5	2.84
Cd	<0.5	<0.25

Source: Adapted from Sepúlveda, B.A.; Pavez O.; Tapia, M. [31] * Alonso etc. [3] (2008, they consider that Salicornia and Sarcocornia belong to a taxonomic complex of highly specialized plants in coastal and saline environments).
